# Severe vivax malaria trends in the last two years: a study from a tertiary care centre, Delhi, India

**DOI:** 10.1186/s12941-020-00393-9

**Published:** 2020-10-30

**Authors:** Monika Matlani, Loick P. Kojom, Neelangi Mishra, Vinita Dogra, Vineeta Singh

**Affiliations:** 1 Department of Microbiology, Vardhman Mahavir Medical College and Safdarjung Hospital, New Delhi, India; 2 Cell Biology Laboratory and Malaria Parasite Bank, ICMR-National Institute of Malaria Research, New Delhi, India

**Keywords:** *Plasmodium vivax*, Vivax malaria, Clinical trends, Disease severity, India

## Abstract

**Background:**

*Plasmodium vivax*, once considered benign species, is recently being recognised to be causing severe malaria like *Plasmodium falciparum*. In the present study, the authors report the trends in malaria severity in *P. vivax* among patients from a Delhi government hospital. The aim of the study was to understand the disease severity and the burden of severe vivax malaria.

**Methods:**

A hospital based study was carried out from June 2017 to December 2018 at a tertiary care centre from Delhi, India. Patients were tested for malaria using peripheral blood smear (PBS) and/or rapid malaria antigen test (RMAT). The severe and non-severe vivax malaria categorization was done as per the WHO guidelines. Sociodemographic, clinic and paraclinical data were collected from patients and their medical records.

**Results:**

Of the 205 patients, 177 (86.3%) had *P. vivax* infection, 22 (10.7%) had *P. falciparum* infection and six (2.9%) had mixed infection with both the species. Out of 177 *P. vivax* cases included in this study one or more manifestations of severe malaria was found in 58 cases (32.7%). Severe anaemia (56.9%), jaundice (15%) and significant bleeding (15%) were the most common complications reported in most of patients, along with thrombocytopenia.

**Conclusions:**

In this study, it is evident that vivax malaria is emerging as the new severe disease in malaria patients, a significant shift in the paradigm of *P. vivax* pathogenesis. The spectrum of complications and alterations in the laboratory parameters in *P. vivax* clinical cases also indicate the recent shift in the disease severity.

## Background

Malaria still remainsa global public health problem. According to the World Malaria Report 2019, India accounts for 4% of all estimated cases of malaria worldwide [1]. India is an endemic region with more than half of the population [698 million] at risk for malaria infections annually where *Plasmodium vivax* is solely responsible for about 50% of the reported malaria cases [2]. A broad spectrum of clinical features may be manifested in humans following the bite of female *Anopheles* mosquitoes with inoculation of the *Plasmodium* species into the blood circulation, leading to asymptomatic parasitaemia, uncomplicated and severe malaria with associated deaths [3]. The outcome of the malaria disease is influenced by various factors like infecting *Plasmodium* species, host immunity and efficacy of treatment [4]. In endemic areas, some of the important risk factors responsible for the severity of disease including the age of the patient, early and frequent relapses with limited access to early diagnosis and treatment, along with prevalence of comorbidities such as bacterial co-infections [5–7].

Historically, *P. vivax* is known to cause benign tertian malaria, but in recent years a change in this trend has been observed with life-threatening symptoms similar to those of *P. falciparum* infections [3]. The most common clinical complications increasingly reported due to severe *P. vivax* malaria are severe anaemia, acute respiratory distress syndrome (ARDS), splenic rupture and acute kidney injury [8]. Several recent reports indicate this significant shift in the paradigm of *P. vivax* [9, 10].

Despite being globally recognised recently, to cause severe disease and mortality, there is still meagreness in estimating the exact burden of severe disease due to *P. vivax*, which poses the need for further studies especially in vivax endemic regions [11, 12]. Here, a prospective observational study was performed to determine the proportion of disease severity, the spectrum of complications and alterations in laboratory parameters in *P. vivax* malaria infections among patients admitted at a tertiary care centre from New Delhi, India.

## Methods

### Study area

This study was conducted in a tertiary care centre situated in New Delhi. The region is hypoendemic for malaria with an annual parasite index of less than 1 [13]. This hospital is a 2400 bedded tertiary care hospital, which caters to patients from whole of northern zone of India with daily outpatient department (OPD) visit of 8000–10,000 patients per day. The monsoon season in Delhi is from July to September months and a surge in malaria cases is observed every year from August to October, the malaria transmission months in this region. According to the data from the National Vector Borne Disease Control Program, *P. vivax* is the predominant species in Delhi State, with prevalence rate > 90% each year [14]. This study was carried out over one and a half years, from June 2017 till December 2018, to include disease transmission period of both the years 2017 and 2018.

### Patients and ethical statement

This study included the patients enrolled in the OPD and tested positive for malaria. Ethical clearance was taken by the Ethics Committee before the beginning of the study [IEC/SJH/VMMC/Project/ 2017/983]. A written informed consent was obtained from each study participant or guardian of the wards, and confidentiality of the participant’s test results was ensured throughout the research. Each study participant was given a unique numerical code in order to streamline the data collection. The inclusion and exclusion criteria of the study were as following:

### Inclusion criteria

- Patients presenting with undifferentiated fever for minimum five days with clinical suspicion of malaria.
- Patients whose blood sample was positive for malaria by peripheral blood smear (PBS) and Rapid malarial antigen test (RMAT), with or without clinical suspicion of malaria.
- Availability of informed written consent and completed performa.

### Exclusion criteria

- Non- availability of informed written consent.
- Patients positive for any other illness other than malaria.

### Sample size

The minimal sample size required for the study was computed using the following formula of Lorentz: N = p (1-p) z^2^/d^2^ where N is the minimal sample size; p is the prevalence of severe vivax malaria (62.9%) reported previously [11]; d the accepted margin of error (d = 0.05) and z the statistic for the desired confidence level (z = 1.96 for confidence at 95%). Thus, a total sample size of 302 individuals was minimally required for this study.

### Diagnosis

A detailed clinical history of the consenting patients was taken and a thorough physical examination was performed. Laboratory investigations were performed to establish the diagnosis of malaria and assess the severity of the disease in these patients.

PBS and RMAT were the diagnostic methods used to detect malaria species in blood samples of patients. The RMAT used in the study is designed to detect two malarial antigens: (1) Histidine rich protein-2 (HRP-2), a protein specifically produced by *P. falciparum*, and (2) Pan malarial lactate dehydrogenase (p-LDH), produced by all malarial species. Giemsa stained thick and thin PBS were examined for the identification of species and counting of malarial parasites, respectively. PBS and RMAT were performed by skilled operators to ascertain the quality of sample. RMAT results were classified as valid (positive or negative) and invalid [15]. The parasite count was calculated from the number of parasitized cells/200 leukocytes in a Giemsa stained thick smears [16]. On the basis of the species identification, the patients were categorized into three groups- 1) *P. vivax* infections*,* 2) *P. falciparum* infections and 3) mixed infections of *P. falciparum* and *P. vivax*. The classification as severe and non-severe vivax malaria was done following WHO guidelines [17]. The patients with uncomplicated malaria were treated following the standard guidelines of national program for malaria treatment, i.e., artesunate–sulfadoxine–pyrimethamine (AS + SP) for 3 days plus single dose primaquine (PQ) on the second day in case of falciparum malaria; chloroquine for 3 days plus PQ for 14 days for vivax malaria; and, AS + SP for 3 days plus PQ for 14 days in case of mixed infection with *P. vivax* and *P. falciparum* [18]. Patients with severe malaria were treated with intravenous artesunate [18].

### Demographical characteristics

Data regarding age, gender and pregnancy status were collected using an ad hoc investigation form conceived for the need of the study.

### Laboratory investigations

The venous blood samples were collected in ethylenediaminetetraacetic acid (EDTA) anticoagulant vacutainer tubes for serological, haematological and biochemical investigations. Complete blood counts, coagulation profile tests, blood glucose, blood urea, serum creatinine, serum electrolytes, serum bilirubin, serum aspartate aminotransferase (AST), serum alanine aminotransferase (ALT) were measured for patients positive for *P. vivax*. Appropriate serological tests were also performed to exclude enteric fever, dengue, chikungunya, scrub typhus and leptospirosis.

### Statistical analysis

Data were keyed into an Excel spreadsheet (Microsoft office 2016, USA) and analysed using the statistical package for social sciences v16 for Windows (SPSS, Chicago, IL, USA). Qualitative and quantitative variables were expressed as frequency (percentages) and mean ± standard deviation (SD), respectively. Confidence interval at 95% (95% CI) of percentages was computed. Parasitemia values were log_10_-transformed before statistical analysis. Pearson’s chi square and Fisher’s exact tests were used to compare percentages, while unpaired sample t test was used to compare mean values. Significance was set at p-value < 0.05.

## Results

**Fig. 1:**
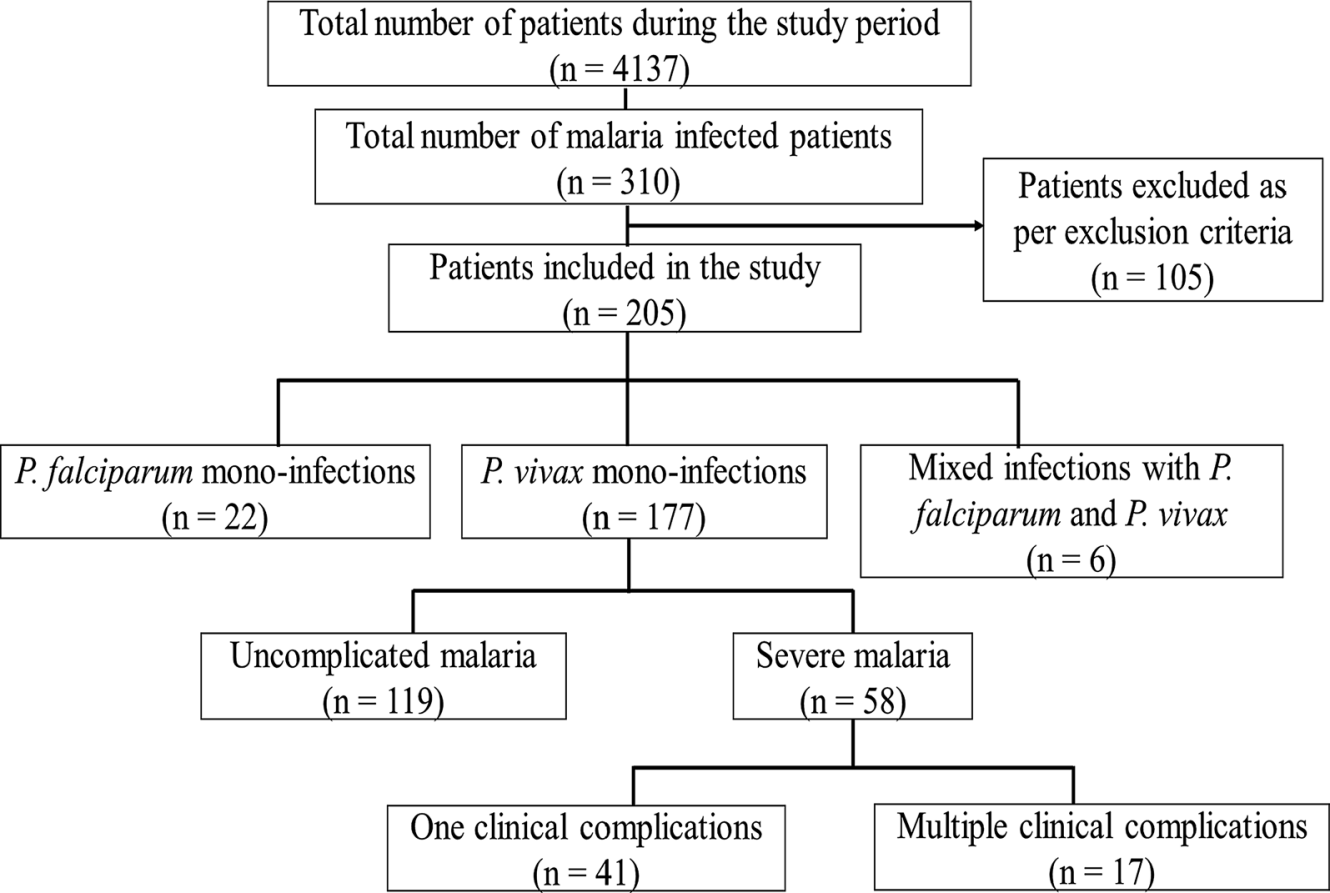
Flowchart of the identification of severe vivax malaria in the study

**Fig. 2:**
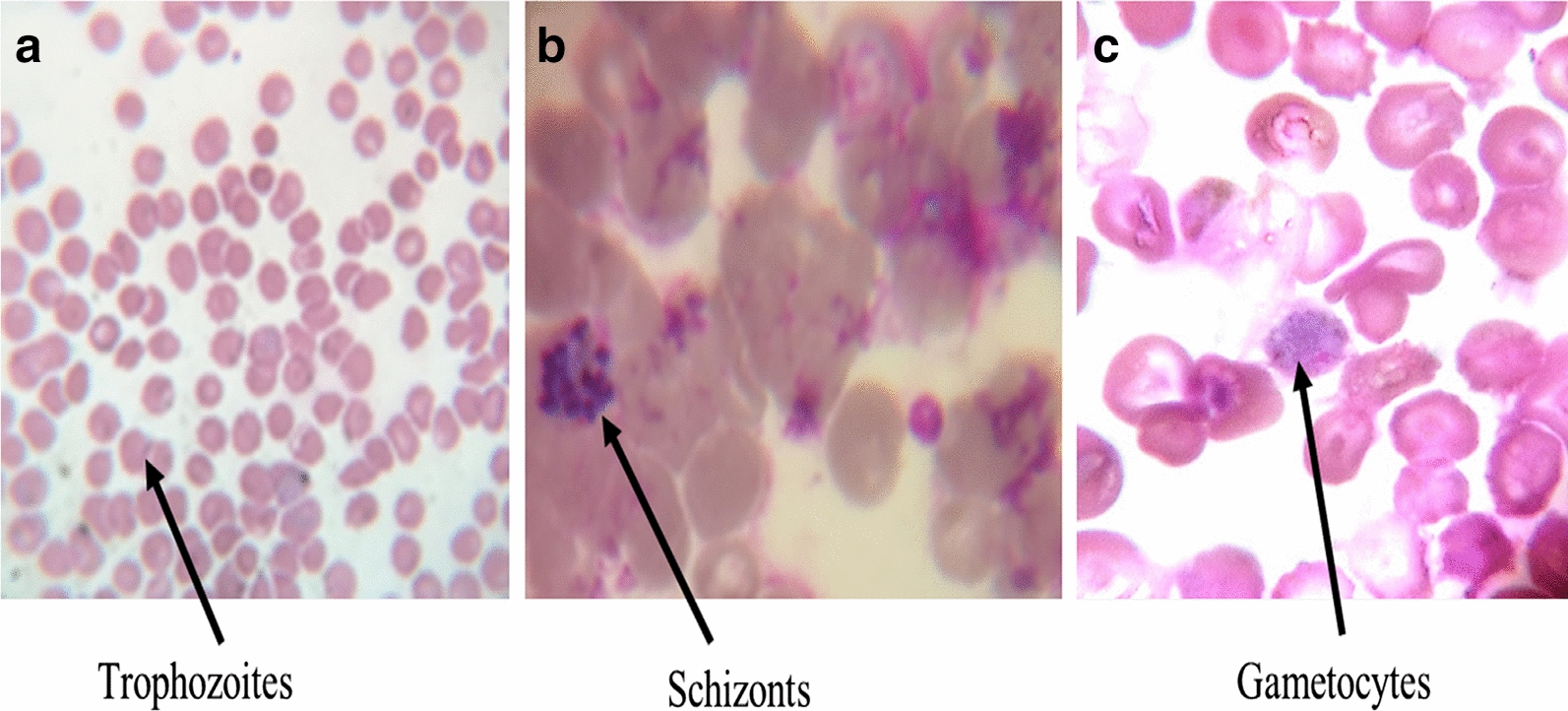
**a** Photographs of trophozoites, **b** schizonts and **c** gametocytes of *P. vivax* blood smears from the study

The onset of malaria case reporting is observed mostly after the raining season annually between the months of August–October (the malaria transmission period) in Delhi and other neighbouring regions. Maximum number of cases were recorded in October 2018 (65 cases) followed by September 2018 (51 cases). A total of 205 confirmed cases of malaria were enrolled in the study, after inclusion criteria were satisfied. Of the 205 patients, 177 (86.3%) were positive for *P. vivax* infection, 22 (10.7%) had *P. falciparum* infection and six (2.9%) showed mixed infections as diagnosed by PBS and RMAT (Fig. 1). There was a predominance of trophozoites and gametocytes in the peripheral blood smears examined, and parasite density ranged from 200–17,800 parasites/µl (Fig. 2). The *P. vivax* group comprised 100 children (< 14 years) and 77 adults. The age of the participants ranged from 2 to 58 years with a median age of 13 years. The number of male patients was higher in both age groups i.e., below 14 years and above 14 years (Table 1).

**Table 1:** Demographic details of the positive *Plasmodium vivax* patients

Age groups	Male	Females	Total
< 14 yrs	58	42	100
> 14 yrs	47	30	77
Total	105	72	177

In the 177 *P. vivax* cases, fever was present in all the patients. Of these, 119 patients (67.2%) had uncomplicated malaria while the remaining 58 (32.7%) were suffering from complicated malaria. In addition, 41 of these 58 patients, had only a single complication, while 17 cases presented > 1 or multiple complications (Fig. 1, Table 2). Thus, the prevalence of severe vivax malaria was found to be 32.7% (95% CI 26.3–40.0%).

**Table 2:** Details of clinical features observed in severe and uncomplicated cases of vivax malaria

Clinical features	Severe malaria (*n* = 58) (%)	Uncomplicated malaria (*n* = 119) (%)	Total number of patients (*n* = 177) (%)	Chi-square (df)	*P*
Hepatosplenomegaly	49 (84.5)	5 (4.2)	54 (30.5)	109.9 (1)	< 0.0001*
Headache	51 (87.9)	9 (7.6)	30 (17.0)	108.8 (1)	< 0.0001*
Pallor	36 (62.1)	5 (4.2)	41 (23.2)	70.2 (1)	< 0.0001*
Seizures (> 2 episodes in 24 h)	8 (13.8)	0 (0.0)	8 (4.5)	14.1 (1)	0.0002*
Altered sensorium	7 (12.1)	0 (0.0)	7 (3.9)	11.7 (1)	0.0006*
Vomiting	12 (20.7)	4 (3.4)	16 (9.03)	12.2 (1)	0.0005*
Jaundice (Bilirubin > 2 mg%)	15 (25.9)	0 (0.0)	15 (8.47)	30.3 (1)	< 0.0001*
Epistaxis	8 (13.8)	0 (0.0)	8 (4.60)	14.1 (1)	0.0002*
Haematuria	3 (5.2)	0 (0.0)	3 (1.70)	3.5 (1)	0.0599
Hematemesis	2 (3.4)	0 (0.0)	2 (1.10)	1.64 (1)	0.2003
Melena	2 (3.4)	0 (0.0)	2 (1.10)	1.64 (1)	0.2003
Hypovolemic shock (SBP < 80 mmHg)	4 (6.8)	0 (0.0)	4 (2.3)	5.5 (1)	0.0184*
Breathlessness	14 (24.1)	1 (0.8)	15 (8.47)	24.3 (1)	< 0.0001*
Pulmonary oedema	8 (13.8)	0 (0.0)	8 (4.60)	14.1 (1)	0.0002*
Rash	4 (6.8)	0 (0.0)	4 (2.26)	5.5 (1)	0.0184*
Decreased urine output	7 (12.1)	0 (0.0)	7 (3.95)	11.7 (1)	0.0006*

The occurrence of clinical manifestations as listed in the table were found to be statistically significant as values for P were mostly < 0.05 between the two groups

Data are presented as frequency (percentage) and mean ± standard deviation

SBP: Systolic blood pressure, df: Degree of freedom

Pearson’s chi-square test was used to compare percentages

^*^: Statistically significant at P < 0.05

**Fig. 3:**
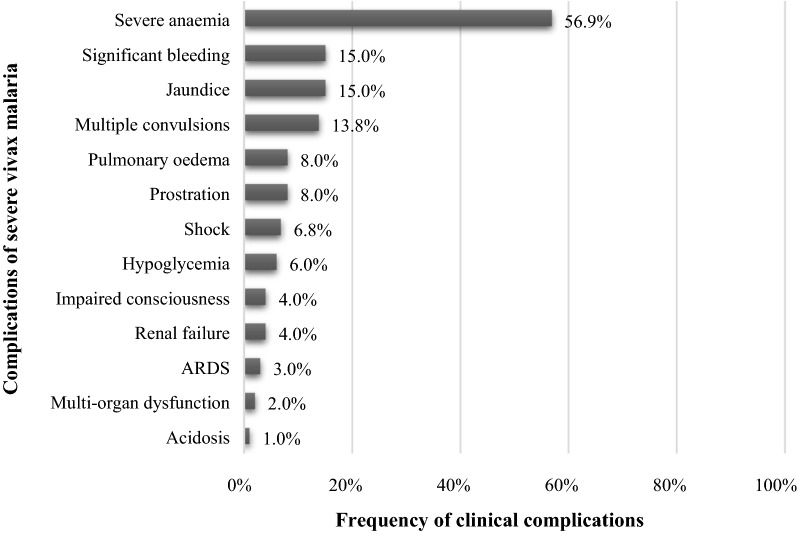
Frequency of different complications observed in patients diagnosed with severe vivax malaria (SVM) during the study period

A total of 13 vivax-related severe complications were found in the studied patients (Fig. 3). Severe anaemia was the most frequent complication found in the study (n = 33, 56.9%) followed by significant bleeding (n = 9, 15%), jaundice (n = 9, 15%) and multiple convulsions (n = 8, 13.8%). Central nervous system (CNS) manifestations in the form of generalised tonic–clonic seizures and altered sensorium were observed in about 4% of the patients which had only 5 children in this group.

All the patients who presented with either seizures or altered sensorium were suspected to be having cerebral malaria attributed to *P. vivax* infection. The analysis of cerebrospinal fluid, computed tomography scan of head, fundoscopy and serum electrolyte studies were also performed to exclude other (bacterial, fungal or viral) CNS infections. Abnormal bleeding was observed in 11 patients (6.2%) in whom epistaxis, hematemesis, haematuria and melena were also observed. On physical examination 50 patients (30.5%) had hepato-splenomegaly, 4 individuals (23.2%) showed pallor, while 15 (47%) were icteric. Apart from these observations, the other clinical findings were oedema, rashes and hypovolemic shock. Clinical details of the patients are shown in Table 2.

The comparative analysis of the prevalence of clinical features in patients diagnosed with severe and uncomplicated malaria revealed statistically significant variations between both groups (P < 0.05). Prevalence of these features were significantly higher in patients with severe complications in comparison to their counterparts diagnosed with uncomplicated malaria (Table 2).

In patients with severe manifestations hepatosplenomegaly was observed to be significantly higher (84.5%, P < 0.0001) in comparison to patients presenting uncomplicated malaria (4.2%). The same pattern was observed for altered sensorium (12.1% vs 0.0%, P < 0.0001), jaundice (25.9% vs 0.0%, P < 0.0001), hypovolemic shock (6.8% vs 0.0%, P < 0.0001), oedema (13.8% vs 0.0%, P < 0.0001) (Table 2).

Details of the laboratory parameters revealed low haemoglobin level in the analysed samples (Table 3). Different degree of anaemia severity was observed in 130 (73.4%) patients, amongst whom the anaemia was graded as mild (Hb 9-11 mg%), moderate (Hb 7-9 mg%) and severe (Hb < 7 mg%) (Table 3). The mean haemoglobin (Hb level) was 4.2 mg% (Table 3). Thrombocytopenia was seen in 116 (65.5%) patients; however none of the patients had any life threatening haemorrhagic episodes. The lowest platelet count recorded was 7000/µl in 1 patient with concurrent dengue infection.

**Table 3:** Laboratory parameters of severe verses uncomplicated cases of malaria

Laboratory parameters	Severe malaria *n* = 58 (%)	Uncomplicated malaria *n* = 119 (%)	Total number of patients *n* = 177 (%)	Chi-square (df)	*P*
Leukopenia (WBC < 4000 cells/µl)	15 (25.9)	10 (8.4)	25 (14.1)	8.4 (1)	0.0037*
Leukocytosis (WBC > 10,000 cells/µl)	6 (10.3)	0 (0.0)	6 (3.4)	9.8 (1)	0.0018*
Severe anaemia	33 (56.9)	0 (0.0)	33 (18.7)	79.5 (1)	< 0.0001*
Moderate anaemia	15 (25.9)	9 (7.6)	24 (13.5)	9.6 (1)	0.0019*
Mild anaemia	38 (65.5)	39 (32.8)	67 (37.9)	15.7 (1)	< 0.0001*
Thrombocytopenia (< 150,000 cells/µl)	52 (89.7)	64 (53.8)	116 (65.5)	20.7 (1)	< 0.0001*
Deranged AST	24 (41.4)	13 (10.9)	37 (20.9)	20.1 (1)	< 0.0001*
Deranged ALT	26 (44.8)	17 (14.3)	43 (24.3)	18.2 (1)	< 0.0001*
Serum bilirubin > 2.5 mg/dL	15 (28.9)	2 (1.7)	17 (9.6)	23.6 (1)	< 0.0001*
Mean Hb ± SD	11.5 ± 1.7	13.4 ± 1.9	–		0.0024*
Mean RBC count (cells/µl)	2.51 × 10^6^	3.41 × 10^6^	–		0.0478*

Data are presented as frequency (percentage) and mean ± standard deviation (SD)

ALT, Alanine aminotransferase, AST, Aspartate aminotransferase, RBC, Red blood cells, df, Degree of freedom, WBC: White blood cells

Pearson’s chi square and unpaired Student t tests were used to compare percentages and mean values respectively

^*^: Statistically significant at P < 0.05

Pancytopenia was seen in 15 cases (8.5%) and packed red cells infusion was given in 2 patients. Deranged liver enzymes and high concentration of serum bilirubin was observed in 24.3% of *P. vivax* patients. Four of the patients who had developed hypovolemic shock with oliguria, loss of consciousness and systolic blood pressure < 80 mmHg were treated with ionotropic agents and fluid replacement also recovered. Two pregnant patients in their third trimester presenting acute febrile illness with anaemia (haemoglobin < 10 gm%) were also included in the study group. Later, during the study intra-uterine deaths were reported in these pregnant patients as neither foetal movements were felt, nor foetal heart sounds were heard and with no other co-morbid conditions. There were 3 cases with dengue co-infections with 1 patient had enteric fever co-infection. No mortality was observed during the course of this study.

All laboratory parameter levels significantly varied according to the severity of vivax malaria (Table 3). Levels of these parameters were significantly higher in severe malaria patients than in uncomplicated malaria patients, with the exception of mean values of Hb and red blood cells (RBC) that were significantly decreased in patients suffering from severe vivax malaria. For instance, the prevalence of leukopenia, severe anaemia and thrombocytopenia were respectively 25.9%, 56.9% and 89.7% in severe patients while they were 8.4%, 0% and 56.8% in uncomplicated malaria patients (Table 3).

The evolution of different haematological features according to vivax parasitemia levels is depicted in Table 4. Statistically significant associations were found for three features viz severe anaemia, mean corpuscular volume (MCV) and mean corpuscular haemoglobin (MCH). The prevalence of severe anaemia gradually increased as a function of parasitemia, from 22.2% in patient group with parasitemia < 100 parasites/µl to 45.5% in patient group with parasitemia > 10,001 parasites/µl (P = 0.0139). Similarly, the prevalence of patients with MCV below normal ranged from 60.5% in patient group with parasitemia < 100 parasites/µl to 100% in patient group with parasitemia > 10,001 parasites/µl P = 0.0351). In contrast, no statistically significant difference was found in total leucocyte count (TLC) between both groups.

**Table 4:** Parasitemia levels of *Plasmodium vivax* and changes in haematological parameters in the studied patients

		Parasitemia range (*P. vivax* mono-infection) (number of parasites/μl of blood)				
Variables	Categories	< 100 [n = 81 (%)]	101–10000 [n = 85 (%)]	> 10001 [n = 11 (%)]	Total n = 177	*P*
Grading of anaemia	Non-anaemic	24 (29.6)	29 (34.1)	0 (0)	53	0.0669
	Mild anaemia	31 (38.3)	34 (40.0)	2 (18.2)	67	0.3716
	Moderate anaemia	8 (9.9)	12 (14.1)	4 (36.4)	24	0.0539
	Severe anaemia	18 (22.2)	10 (11.8)	5 (45.5)	33	0.0139*
Thrombocytopenia	Presence	52 (64.2)	57 (67.1)	7 (63.6)	116	0.9185
Changes in TLC	Leukopenia	10 (12.3)	15 (17.6)	0 (0)	25	0.2357
	Normal TLC	69 (85.2)	66 (77.6)	11 (100)	146	0.1275
	Leukocytosis	2 (2.5)	4 (4.7)	0 (0)	6	0.5916
Changes in MCV	Below normal	49 (60.5)	56 (65.9)	11 (100)	116	0.0351*
	Normal	7 (8.6)	6 (7.0)	0 (0)	13	0.5827
	Above normal	25 (30.9)	23 (27.1)	0 (0)	48	0.0928
Changes in MCH	Below normal	28 (34.6)	25 (29.4)	0 (0)	53	0.0627
	Normal	46 (56.8)	55 (64.7)	8 (72.7)	109	0.0002*
	Above normal	7 (8.6)	5 (5.9)	3 (27.3)	15	0.0564

Data are presented as frequency (percentage) and mean ± standard deviation

TLC, Total leucocyte count, MCV: Mean corpuscular volume, MCH: Mean corpuscular Hb

Fisher’s exact test was used to compare percentages

* Significance was at P-value < 0.05

## Discussion

The clinical course of malaria depends on several host and parasite-related factors and it manifests with a multitude of signs and symptoms. The disease spectrum progresses from stage of asymptomatic parasitemia to uncomplicated malaria, severe malaria and leading to death in some cases. Severe or complicated malaria is more often associated with *P. falciparum* infection, in which increased sequestration of RBC in the microvasculature and massive haemolysis lead to complications like cerebral malaria (CM), renal dysfunction, hepatic dysfunction and ARDS [19]. The biological basis of development of CM is well described for *P. falciparum* through the cytoadherence phenomenon, whereas little is known about molecular basis of CM due to *P. vivax* [20]. There are very few studies which indicate the ability of *P. vivax* to also elicit cytoadherence [21, 22]. Beyond these parasite sequestration-related complications, non-sequestration related complications, including anaemia and thrombocytopenia are also seen in falciparum malaria [23]. Recently, reported severe manifestations of *P. viva*x infections include CM, hepatic dysfunction, acute renal injury, severe anaemia, ARDS, splenic rupture and multiple organ failure [1]. Various studies from other countries such as Ethiopia, Papua New Guinea, Brazil, Indonesia and recently India have also described this increasing trend in the ability of *P. vivax* to elicit severe complications [1].

*P. vivax* accounts for one third of all malaria cases detected in India, with high prevalence in the urban areas [2]. In the present study, we report 177 febrile cases (86.3%, 177/205) of vivax malaria diagnosed by PBS and RMAT where children and male population were more commonly infected. The low level of anti-*P. vivax* immunity observed frequently in children may explain a higher burden of malaria infection in them as found in *P. falciparum* [24]. A total of 58 (32.7%) severe vivax cases were found in the present study. This value is globally lower than one of the previous studies conducted in the same area and from other Indian areas (Table 5) [11, 12, 25–32]. In contrast, this prevalence value is higher than one of studies conducted outside India, especially from African (Ethiopia), Asian (Indonesia, South Korea) and Latin American (Papua New Guinea) countries [33–36].

**Table 5:** Summary of severe *Plasmodium vivax* malaria and complications reported from different regions of India

Study	Year of data collection	Region	No. of *P. vivax* malaria cases	Prevalence of severe vivax malaria	Complications observed
Kochar et al. [25]	2009	Rajasthan	1091 malaria cases 456 (41.8%)- *P. vivax*	40 (8.8%)	Jaundice-57.5%
					RF-32.5%
					Cerebral malaria-12.5%
					Thrombocytopenia-12.5%
					ARDS-10%
					Shock-7.5%
					Hypoglycaemia-2.5%
Yadav et al. [11]	2011	Delhi	147 malaria cases 89 (60.5%)- *P. vivax*	56 (62.9%)	Abnormal bleeding- 30.3%
					Impaired consciousness-20.2%
					Severe anaemia-17.4%
					Jaundice-13.5%
					Multiple convulsions-10.1%
					RF-6.1%
					ARDS-2.2%
					Metabolic acidosis and Hypoglycaemia-1.1%
					Thrombocytopenia-81.2%
Singh et al. [12]	2012–2013	Uttar Pradesh	401 malaria cases 185 (46.1%)-*P. vivax*	-	Icterus-32.9%
					Severe anaemia-20.5%
					Cerebral malaria-15.1%
					Hypoglycaemia-2.1%
					Thrombocytopenia-51.3%
Chery et al. [26]	2012–2015	Goa	1088 malaria cases 838 (77%)- *P. vivax*	56 (78.9%)	ARDS-42.9%
					Jaundice-41.1%
					RF-19.7%
					Shock-7.1%
					Pulmonary oedema-6.9%
					Severe anaemia-2.8%
					Abnormal bleed- 1.4%
Gehlawat et al. [27]	2013	Haryana	47 children with malaria	18/35 (51.4%)	Impaired consciousness-50.9%
					Convulsions-44.4%
			35 severe malaria cases enrolled		Jaundice-27.8%
					Severe anaemia-27.8%
					Shock-16.7%
Kumari et al. [28]	2014	Maharashtra	50 children with *P. vivax* malaria	13 (26%)	Abnormal bleed- 36.5%
					Impaired consciousness- 30.8%
					Severe anaemia- 23%
					ARDS-15.4%
					Shock-15.4%
					Multiple convulsions-7.7%
					Thrombocytopenia-94%
Meena et al. [29]	2017	Rajasthan	55 children with malaria 32 (58.2%)-*P. vivax*	26 (60.4%)	Prostatration-49%
					Abnormal bleed-30.9%
					Severe anaemia27.3%
					RF-20%
					Shock-16.7%
					Convulsions-12.7%
					Pulmonary oedema- 2.7%
					Thrombocytopenia-70.9%
Mathews et al. [30]	2019	Delhi	150 cases of *P. vivax* malaria	63 (42%)	Jaundice-36%
					ARDS-12.7%
					Abnormal bleed-8.67%
					Metabolic acidosis- 5.33%
					RF- 3.33%
					Severe anaemia- 2.67%
					Convulsions- 0.7%
					Thrombocytopenia-86.64%
Anvikar et al. [31]	2016–2017	Gujarat	50 patients with *Plasmodium* infection	30 (73.2%)	Prostration-90%
					Multiple convulsions-70%
					Jaundice-33.3%
					Severe anaemia-3.3%
					Abnormal bleeding-3.3%
					Shock-3.3%

RF- Renal failure, ARDS- acute respiratory distress syndrome

Severe anaemia was the most frequent complication observed in the present study. This finding is in line with previous studies, which showed that this complication is frequently observed in severe *P. vivax* malaria (Table 5). Severe anaemia and haemostatic complications occur due to the potential ability of *P. vivax* to lyse not only the infected RBC, but also the normal RBC as well [36, 37]. Umbers et al. [38] and Riken et al. [39] have established that vivax-associated microvascular dysfunction along with maternal anaemia may cause deleterious utero-placental haemodynamic instability and foetal growth restriction. Severe anaemia is one of the leading cause of deaths in pregnant and children women [23]. Also in our study, intra-uterine deaths were observed in 2 of the *P. vivax* infected pregnant patients, confirming the implication of *P. vivax* in complications of malaria in pregnancy. This finding is consistent with previous studies [40–42].

In this study, the percentage of thrombocytopenic patients was 89.7% (52/58) in severe malaria group, and 53.8% (64/119) in uncomplicated malaria group as reported also from other regions in the country (Table 5). Thrombocytopenia (65.5%, 116/177) was reported in substantial number of patients. Jaundice (15%, 30/177) and abnormal bleeding (15%, 30/177) were the second most common severe manifestations. Multiple convulsions were also observed in 4.5% of cases. Other studies from different countries worldwide have also reported similar manifestations among severe vivax malaria cases [11]. However, renal dysfunction and ARDS were not observed in the present study, but has been observed in previous studies from India and outside India (Table 5).

Anaemia was observed in all three categories of mild, moderate and high parasitemia patients, but a significant relationship between haematological changes and parasitemia levels could not be established, unlike previous studies [26]. This could be attributed to low transmission of malaria in our region, presence of co-morbidities or a poor nutritional status of the patients. Similarly, both leukopenia and leucocytosis were observed in all categories of parasitemia. It has earlier been observed that alterations in haematological parameters in the course of a falciparum malaria infection, such as anaemia, vary with the level of malarial endemicity, background haemoglobinopathy, nutritional status, demographic factors, and malaria immunity [43]. This pattern is similar to the one observed in vivax-induced anaemia [37].

Considering the re-emergence of *P*. *vivax* malaria in several areas, our observations provide an insight into increased number of clinical issues related to the severity of *P*. *vivax* malaria (Table 5). Biological mechanism underlying the pathogenesis in *P. vivax* infections is poorly understood, in addition; there is paucity of sufficient data on this aspect. A further large-scale study is required to determine the underlying pathogenesis of the severity of the disease, and the degree to which it is related to the emerging multidrug resistance in *P. vivax* malaria. Thus, there is an urgent need to re-examine the clinical spectrum and burden of *P*. *vivax* malaria in our settings, so that adequate control measures can be implemented against this disease. Despite effective reduction in the transmission, there is an increase in the number of complicated *P. vivax* cases which significantly contributes to the severity and morbidity in malaria and every effort should be made to reduce or eliminate the malaria burden. We should also target towards reducing infections due to *P. vivax* along with *P. falciparum* in regions where both these species coexist.

## Limitations

This study has few limitations. First, we were unable to make comparison of severity signs between *P. falciparum* and *P. vivax* due to low sample size of *P. falciparum* cases in the present study. Second, comorbidities such as malnutrition eliciting haematological disorders along with the aetiology of anaemia cases were not investigated. Finally, study findings drawn from low sample size of vivax patients attending this heath facility cannot reflect the whole malaria situation in the Delhi State.

## Conclusions

It is notable that *P. vivax* is emerging as an important cause of malaria and morbidity in several endemic and non-endemic regions in India. In addition, the present study demonstrates the important role of *P. vivax* in complications observed in malaria disease pathogenesis. Considering the observed trend in the disease severity from the reported study there is need to strengthen the control programs targeted towards disease severity due to *P. vivax*.

## Data Availability

All datasets on which the conclusions of the research rely are presented in this paper. However, data is available from the corresponding author on reasonable request.

## Abbreviations

ALT: Alanine aminotransferase
ARDS: Acute respiratory distress syndrome
AS: Artesunate
AST: Aspartate aminotransferase
CI: Confidence interval
CNS: Central nervous system
df: Degree of freedom
Hb: Haemoglobin
MCH: Mean corpuscular haemoglobin
MCV: Mean corpuscular volume
PBS: Peripheral blood smears
PQ: Primaquine
RBC: Red blood cells
RF: Renal failure
RMAT: Rapid malaria antigen test
SBP: Systolic blood pressure
SD: Standard deviation
SP: Sulfadoxine-Pyrimethamine
TLC: Total leucocytes count
WBC: White blood cells
WHO: World health organisation
